# Wearable Surface Electromyography System to Predict Freeze of Gait in Parkinson’s Disease Patients

**DOI:** 10.3390/s24237853

**Published:** 2024-12-09

**Authors:** Anna Moore, Jinxing Li, Christopher H. Contag, Luke J. Currano, Connor O. Pyles, David A. Hinkle, Vivek Shinde Patil

**Affiliations:** 1 Precision Health Program, Michigan State University, East Lansing, MI 48824, USA; moorea57@msu.edu; 2 Department of Radiology, Michigan State University, East Lansing, MI 48824, USA; 3 Institute for Quantitative Health Science and Engineering, Michigan State University, East Lansing, MI 48824, USA; jl@msu.edu (J.L.); contagch@msu.edu (C.H.C.); 4 Department of Biomedical Engineering, Michigan State University, East Lansing, MI 48824, USA; 5 Department of Microbiology, Genetics and Immunology, Michigan State University, East Lansing, MI 48824, USA; 6 Applied Physics Laboratory, Johns Hopkins University, Laurel, MD 20723, USA; luke.currano@jhuapl.edu (L.J.C.); connor.pyles@jhuapl.edu (C.O.P.); 7 Ohio Health Riverside Methodist Hospital, Columbus, OH 43214, USA; david.hinkle@ohiohealth.com; 8 EXOForce Robotics, Arlington, VA 22209, USA

**Keywords:** freeze of gait, Parkinson’s disease, electromyography, prevention

## Abstract

Freezing of gait (FOG) is a disabling yet poorly understood paroxysmal gait disorder affecting the vast majority of patients with Parkinson’s disease (PD) as they reach advanced stages of the disorder. Falling is one of the most disabling consequences of a FOG episode; it often results in injury and a future fear of falling, leading to diminished social engagement, a reduction in general fitness, loss of independence, and degradation of overall quality of life. Currently, there is no robust or reliable treatment against FOG in PD. In the absence of reliable and effective treatment for Parkinson’s disease, alleviating the consequences of FOG represents an unmet clinical need, with the first step being reliable FOG prediction. Current methods for FOG prediction and prevention cannot provide real-time readouts and are not sensitive enough to detect changes in walking patterns or balance. To fill this gap, we developed an sEMG system consisting of a soft, wearable garment (pair of shorts and two calf sleeves) embedded with screen-printed electrodes and stretchable traces capable of picking up and recording the electromyography activities from lower limb muscles. Here, we report on the testing of these garments in healthy individuals and in patients with PD FOG. The preliminary testing produced an initial time-to-onset commencement that persisted > 3 s across all patients, resulting in a nearly 3-fold drop in sEMG activity. We believe that these initial studies serve as a solid foundation for further development of smart digital textiles with integrated bio and chemical sensors that will provide AI-enabled, medically oriented data.

## 1. Introduction

Parkinson’s disease (PD) is a common, multi-faceted neurodegenerative disorder for which no curative or disease-modifying treatment is available [1], and with an annual incidence in North America of approximately 60,000 to 95,000 among adults ages 45 and older, PD remains a significant healthcare problem. Dopamine replacement pharmacotherapy is effective for tremor, rigidity, and bradykinesia symptoms but not for most gait symptoms (particularly at advanced stages). Freezing of gait (FOG) is a highly disabling yet poorly understood paroxysmal gait disorder affecting the vast majority of patients in the late stages of PD [2,3,4]. The prevalence of FOG in PD patients is unclear and varies widely from as low as 5% [5] up to 85.9% [6]. One recent study placed this number at an average prevalence of 35.8% but cautioned that it varies significantly with different disease durations and severities [7]. Typically, a FOG episode is very brief, often lasting only 1–2 s, although it could last up to 30 s [8]. It is most commonly experienced during turning and step initiation but also occurs when faced with spatial constraint, stress, and distraction [9]. FOG causes falls, leading to injuries, a future fear of falling, and loss of independence [10]. Furthermore, the functional impact of FOG is independently linked to a reduced health-related quality of life (HRQoL), irrespective of other general disease severity measures [4]. 

Falling is one of the most disabling features of FOG in PD and could result in a hip fracture, which is increased 2.8 times in women and 5.3 times in men with PD when compared with age- and gender-matched controls [11]. In addition, fear of falling leads to anxiety [12], reduced mobility, and a subsequent development of weakness from muscular deconditioning [13], a reduction in general fitness, loss of independence, and increased risk of hospitalization [14,15], leading to nursing home admission [16] and reduced survival [17]. Since freezing of gait can be affected by anxiety, if a person feels rushed (for example, under a time constraint to board an elevator before the doors close), the risk of freezing is increased. 

The complications of FOG impact not only the affected patients and their families but also the healthcare system, in general, by increasing broader societal costs [18,19,20]. One recent study has estimated the costs of fatal and nonfatal falls to total approximately USD 50 billion [21]. Medicare spending attributable to nonfatal older adult falls totaled USD 28.9 billion. Almost 99% of this cost was attributable to health care for nonfatal falls. These cost trends are significant and of general concern because 75% of the cost of older adult falls is financed through public health insurance programs such as Medicaid that are already financially stressed [22]. In the absence of reliable and effective treatment for advanced PD, alleviating the consequences of FOG in PD remains an unmet clinical need.

Generally, falls due to FOG occur during normal daily activities. Therefore, user-friendly methods of fall prediction and prevention that can be deployed at home have been a focus of PD researchers. The current evaluation methods for FOG prediction and prevention include the *Timed up and Go* test (TUG; [10,12]), *Unified Parkinson’s Disease Rating Scale* (UPDRS; [10,23]), and *Freezing of Gait Questionnaire* (FOG-Q; [6,24]). Unfortunately, most of these measures have limited specificity and sensitivity for identifying patients with an elevated risk of falling, ref. [25] cannot provide real-time readouts, and are not sensitive enough to detect functionally relevant changes in balance and walking control in PD patients with mild to moderate disease severity [7,26,27,28]. Mechanically averting FOG is one area where some progress has been made. A recent study [29] reported on a wearable garment that uses cable-driven actuators and sensors, generating assistive moments in concert with biological muscles. Testing of this garment in one person (a 73-year-old male with PD) demonstrated the elimination of FOG episodes during walking. However, this apparel does not have the capability to predict FOG. Predictive indicators would give the patient the time and opportunity to be prepared and hopefully prevent the fall. Another study [30] published the use of wrist-worn sensors with IMUs for detecting FOG in PD patients. Although this would be useful information, the ability to predict and not just detect FOG would offer greater benefit to the patient. The use of sensors integrated into leggings, such as in EXOForce garments that provide direct contact with affected muscles is an approach that would have the benefit of prediction that is not possible with sensors worn on the wrist [30]. At present, there is currently no method that can be used in real time to detect and then prevent FOG, making predictability one of the main challenges. 

While standalone sensors (such as inertial measurement units (IMUs) and surface electromyography (sEMG) for measuring gait velocity and muscle activity, respectively) have been developed, they have remained unwieldy in their design. Effective use of IMUs and sEMGs for FOG profiling and prediction requires integration into a user-friendly, readily deployed, single wearable system that is comfortable and has seamless harmonization of the data with a readout that can help prevent FOG. Despite being widely desired by the PD research community [31], integrated portable systems that have sufficient convenience for at-home daily use have not yet been developed. Many systems-based attempts [32,33,34] have used different classes of sensors that were either strapped on or adhered to discrete sites on the body, making them uncomfortable, obtrusive, and not washable. sEMG sensing has, to date, relied on the use of gel-based medical electrodes that are wired and require gel application and cleanup, which significantly restricts the frequency of use and the number of electrodes that can be integrated.

There are a handful of major market players (for example, Sensoria Health, Redmond WA, USA and Myontec, South Pasadena, FL, USA) operating in the wearable sensor space for PD symptom detection, but none of them has produced a fully integrated system capable of synchronizing the data and, more importantly, alerting patients in advance of (or automatically treating) an upcoming FOG episode. To fill this gap and to improve the overall multiparametric profile of patients with FOG at risk of falling, we developed a wearable sEMG recording system consisting of a pair of shorts and two calf sleeves embedded with screen-printed electrodes and stretchable traces that are capable of picking up electromyography activity from lower limb muscles. Integrating the soft electrodes and traces with wearable cloth textiles offers high-fidelity and highly comfortable EMG recording without interrupting daily activities. To benchmark these garments for performance, we first tested them in controlled exercise routines in healthy volunteers with both upper- and lower-body garments. These tests showed an excellent correlation between EMG burst activity and period of pause. Next, preliminary testing of these garments in PD patients with FOG produced the initial time-to-onset commencement that persisted > 3 s across all patients, resulting in a nearly 3-fold drop in sEMG activity. Our user satisfaction study showed that all patients expressed satisfaction with the comfortability as part of the usability study performed after the completion of the testing. The main contribution of our research to the field is that we showed that it is possible to predict FOG episodes and alert patients who are prone to falls, therefore solving the unmet clinical need for injury prevention. We acknowledge that testing in a large cohort of PD patients is needed to fully validate our approach, which we plan to do in the near future. We also believe that these initial studies serve as a basis for further development of smart digital textiles with integrated bio and chemical sensors that provide AI-enabled, medically oriented data not only for healthcare but also for athletics and military applications. 

## 2. Materials and Methods

### 2.1. Garment Assembly

**Figure 1:**
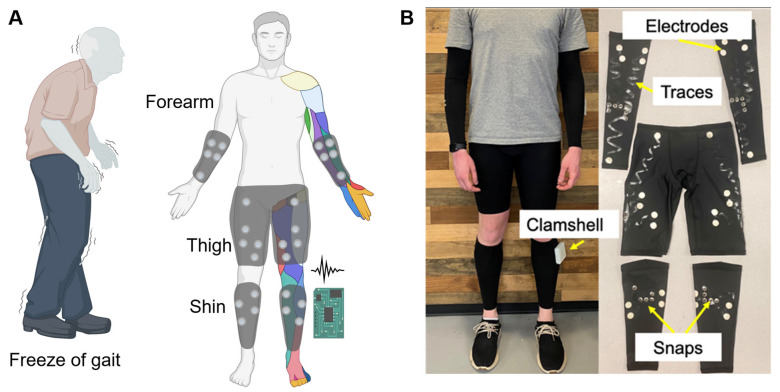
(**A**) Illustration of the wearable multi-channel EMG to predict freeze of gait in Parkinson’s disease patients. (**B**) The soft wearable garments are embedded with EMG sensors.

As shown in Figure 1A, the lightweight garments (n = 10) were assembled by Harbor Designs and Manufacturing, LLC (Baltimore, MD, USA), contracted by EXOForce Robotics in consultation with the Johns Hopkins University Applied Physics Laboratory, Laurel, MD, USA (JHU/APL), as described in [35]. Briefly, the base textile (M-200, Sportek, Commerce, CA, USA) was composed of 92% polyester and 8% spandex, which exhibits required stretchability and wicking ability/moisture management. Stretch athletic base textiles, conductive commercial stretch material (CCSM) (A321, Less EMF), single- and double-sided (TE-21C, Dupont Intexar, Dupont, Wilmington, DE, USA) thermoplastic polyurethane (TPU) films with integrated melt adhesive, patterned Kapton flex connectors (300 µm thickness, Dupont, Wilmington, DE, USA), and foam cushions (1454120-1540, Online Fabric Store) were cut to an appropriate size and shape with a laser-cutter (Fusion Pro 32, Epilog Laser, Golden, CO, USA). Nickel-plated snap fasteners (116-65, Dritz, Spartanburg, SC, USA) were added with a snap application tool (16-P, Dritz). Throughout the process, heat-press steps were performed for 23 s at 290 °F [35]. The characteristics of the products were similar to those reported previously [35]. For this minimal viable product (MVP) phase, we only developed two sizes (medium and large) based on conventional athletic gear sizing. The biosensor electrodes (3/4 in diameter, n = 2/muscle group) were screen-printed using proprietary ink blends and feature a flexible and stretchable base film with a carbon underprint to reduce moisture transfer. A carbon overprint adds an additional layer of wear resistance. We determined the Intexar™ stretchable traces (DuPont) to be our preferred conductors/traces for this solution due to their conductive performance (maintained for nearly 50 gentle washes). The garments were assembled using heat application to adhere the biosensors and traces to the base layer garment. A sealant was then applied to protect the traces from long-term wear and tear. A pattern of biosensor electrodes was placed over all the major muscle groups in the upper and lower leg: hamstrings, quadriceps, tibialis anterior (shin), and gastrocnemius (calf) muscles, and were connected via conducting traces that originate from these electrodes and terminate in nickel-plated snap fasteners that interface with the garment electronics.

### 2.2. Electronics Assembly

The garment’s electronics consisted of an electrophysiology amplifier chip (ADS1294, Texas Instruments, Santa Clara, CA, USA), an ESP32-WROOM-32D microcontroller (Espressif Systems, Shanghai, China) that features a Bluetooth 4.2 as well as the Wi-Fi 802.11b/g/n module, and low-power batteries among other relevant components encased in a removable 3D-printed clamshell and connected via metal snaps (Figure 1B). The amplifier was used to receive, amplify, and digitize the sEMG signals collected by the garment. The onboard firmware was used to process the data and transmit it wirelessly to a computer-based MATLAB 2020a visualization interface.

### 2.3. Garment Testing in Healthy Subjects

Before embarking on testing the garments in PD patients, the systems were tested in healthy volunteers (n = 6). Specifically, we tested EMG muscle activity in a biceps muscle group in test subjects who were asked to do a specific routine: 10 s pause, 30 s of bicep curls, 30 s pause, and bicep curls until they became fatigued with 35 lb dumbbells. The muscle activity was gathered from the right arm sleeves. The training load was normalized against an isometric threshold.

**Figure 2:**
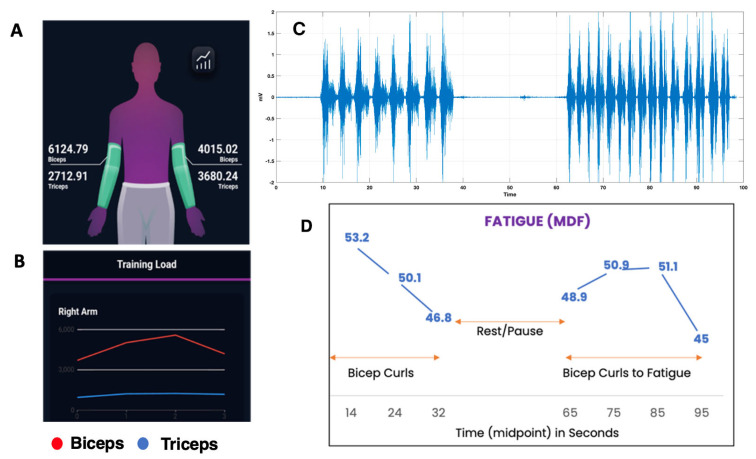
Training load and fatigue data from the testing of garments in healthy subjects. (**A**,**B**) Visual snapshots of the software app are shown for demonstration purposes only. They showcase how the app can be used to represent the user workout numerically and graphically in various muscle groups. (**C**) Shown are the bursts of EMG activity corresponding to bicep curls with period of pauses at the onset and between two repetitions. (**D**) Median Frequency (MDF) plot, a frequency-domain feature used to assess muscle fatigue corresponding to the bicep curls in (**C**).

Calculations for both arms are shown in Figure 2A. The training load is shown graphically over the duration of the workout in Figure 2B. Our results indicate that the soft screen-printed electrodes can obtain high-fidelity sEMG recordings.

### 2.4. Parkinson’s Disease Subject Study

Patients with PD (n = 3) were recruited to the study by the Movement Disorders Clinic at the OhioHealth Riverside Methodist Hospital (Columbus, OH, USA). Written informed consent was obtained from each subject. The study was designed to study sEMG activity underlying gait in PD patients and obtain usability feedback on the garment itself. The patients were instructed to wear shorts and to walk through an obstacle course similar to the Turning and Barriers Course Figures of 8 (TBC-F8) [36]. This mimicked a real-world situation where they encounter objects, slow their speed, and walk around them, all of which have the potential to precipitate FOG episodes. The electromyography (sEMG) activity from the lower limb muscles was picked up by the electrodes placed on the quadriceps and hamstrings, conducted through soft conducting traces, and processed through a sophisticated electronics infrastructure to amplify and filter relevant electrical signals. All raw sEMG signals were passed through a 15–450 Hz bandpass filter and subjected to a sliding window calculation with a 200 ms window length and 10–50 ms step size (depending on the computing power). 

The acquisition of the data was conducted in real time by video recording at a video frame rate (33 frames/s). The patients were videotaped from the waist down to track their motion and to validate the exact timestamp of their freeze commencement. In parallel, the data from the sEMG sensors were visualized in a MATLAB-based sEMG data visualization tool. The research staff performed a video recording of the patients as they navigated the obstacle course and timestamped all major events (the start, end, onset of FOG episode, end of FOG episode, etc.) Each of the timestamps was reconciled with the timestamps on the visualization tool to address latency issues and ensure a complete alignment of start times. Given this is a pilot study with prototype garments, data processing was done after completion of the study. In addition to testing in the obstacle course, an informal usability study using a Likert scale (1–5, 1 being the lowest level of satisfaction and 5 being the highest level of satisfaction) was performed where the patients were asked to provide feedback to the following questions:

1. Do you like the garments?
2. Are they comfortable?
3. Would you be confident using these garments at home by yourself?

The patients’ answers were collected and analyzed. All studies involving human subjects were reviewed in accordance with the ethical guidelines and approved by the Institutional Review Board (IRB) at the OhioHealth Riverside Methodist Hospital.

### 2.5. Statistical Analysis

A one-way analysis of variance (ANOVA) was conducted (Matlab, Mathworks Inc., Natick, MA, USA) to determine statistically significant differences between the level of effort and fatigue values between active and pause states.

## 3. Results

Initial testing of the garment was performed by testing EMG muscle activity in a biceps muscle group in healthy volunteers. The results of the testing of the muscle activity in a biceps muscle group collected from the right arm sleeve are shown in Figure 2C. We observed a remarkable correlation between EMG burst activity during each corresponding set of bicep curls and periods of pause. Finally, the decreasing median frequency values (fast Fourier transform of the data) are indicative of emerging fatigue in the second set of curls (Figure 2D). 

**Figure 3:**
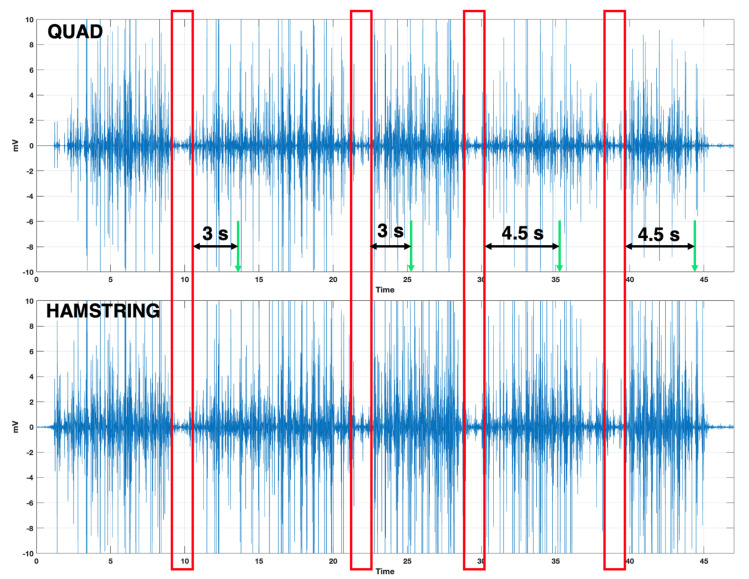
Patterns of EMG activity in quadriceps and hamstring muscles (PD patient). The duration of the lowered EMG activity is indicated by the red windows, while the green arrows denote the onset of the FOG episode. The times shown (seconds) highlight the timing between the lowered EMG activity and the onset of the FOG episode.

Having shown that our garments are capable of registering muscle activity with high accuracy, we next embarked on testing shorts in patients with Parkinson’s Disease FOG. During the course of our study, all three patients encountered a total of eight FOG episodes, as shown in Figure 3, representing a sample dataset from one of the patients. The blue lines indicate sEMG activity in the quadriceps and hamstring muscles of the right leg in millivolts (mV) as a function of time. The red boxes indicate times when there was a loss of sEMG signals, which correlated to the four episodes of FOG that this patient experienced. These data were recorded over a ~50 s session. The red blocks highlight noticeable drops/changes in sEMG data patterns. The time stamps (3–4 s) represent the *time to onset* or the number of seconds *before* their FOG episode commenced. Significant drops in muscle activity in the thigh muscles were recorded, as well as in anterior and calf muscles, though the latter were less consistent. Noteworthy, the tibialis anterior muscles have previously been implicated in FOG episodes by another research group [34], thereby warranting further investigation. 

**Figure 4:**
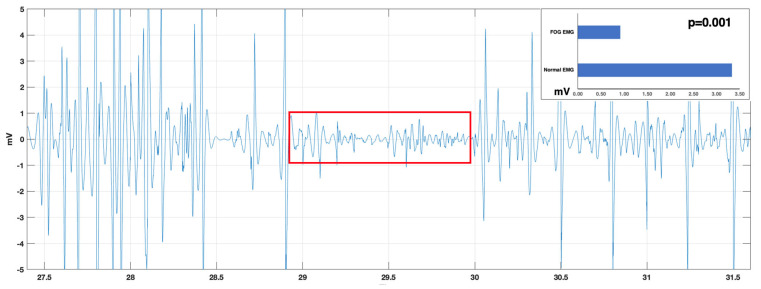
(Zoomed in from Figure 3) EMG patterns demonstrate a 3-fold drop (*p* = 0.001) in EMG activity prior to FOG compared to normal EMG values. This drop in activity commences 3–4.5 s before the onset of a FOG episode. The duration of the lowered EMG activity prior to FOG is indicated by the red window.

As shown in Figure 3, even though there was a noticeable variability in the initial time-to-onset commencement, it persisted > 3 s across all three patients. The upcoming FOG episodes (indicated by the green arrows) were preceded by the lower EMG activity (red windows) for 3 or more seconds, providing the patient with time to react. Furthermore, we observed a nearly 3-fold drop in sEMG activity (*p* = 0.001, n = 8), as shown in Figure 4. While normal sEMG activity usually oscillates between ±3 mV [37], sEMG activity preceding a FOG episode dropped to ±1 mV in our study. This decrease in sEMG activity compared to normal EMG values commences 3–4.5 s before the onset of a FOG episode. Timestamps on the sEMG data and the video feed were repeatedly validated to ensure that there was no time lag between the events. These noticeable drops in sEMG patterns coupled with a consistent time-to-onset observation, serve as a foundation for our further studies that such a signal drop might be used as an alert system for Parkinson’s patients. With modifications, we expect significant performance improvements with increased sensitivity and reduced noise, in addition to improvements in convenience and wearability.

In addition to performing initial testing, we also obtained usability feedback on the garment from the participants. All the patients gave the highest level of satisfaction (“5” on a Likert scale) to the questions regarding the likability of the garments, comfort during wearing, and confidence in using them at home. This positive feedback is extremely important, as it provides assurance of the need for developing these garments further.

## 4. Discussion

Patients in the mid to late stages of Parkinson’s Disease often experience a freeze of gait, defined as a brief, episodic absence or marked reduction in the forward progression of the feet despite the intention to walk [38]. The postural imbalance resulting from a FOG episode presents a risk of falling that can lead to injuries, including fractures, broken spines, and other debilitating injuries that result in ER admissions. Smart textiles with embedded, well-integrated muscle, movement, and biochemical sensors that are interpreted by machine learning (ML)-enabled algorithms to generate medically relevant data would be highly advantageous for predicting FOG episodes in patients with PD. While aversion to FOG has recently been shown in one patient [29], this study did not address the issue of predictability of the episodes, which still remains an unmet need as a first step in injury prevention. To the best of our knowledge, devices affording an actual prediction of the FOG episodes are still lacking. To address this gap, we are developing comfortable, washable, and unobtrusive lower-body leggings embedded with multiple classes of motion and muscle activity sensors that will identify a temporal window of opportunity to intervene before the onset of a FOG episode. Here, we report on the development and evaluation of a first generation of a soft and wearable garment for recording sEMG data. These were tested first in healthy individuals and then in patients with Parkinson’s Disease. We show that the system can be used to monitor the sEMG with high quality and comfort and predict the freezing of gait in persons with Parkinson’s disease. Thus, the technology may lead to a system that can detect and then alert a person to, or even treat, an impending FOG episode. This would significantly improve life quality and disease management for these patients through real-time monitoring and active alerts. As this was the first reported study, we need to acknowledge its limitations. While the observed EMG data were highly accurate, some inconsistencies were noted, most likely due to poor electrode contact or poor fit. By improving the electrodes’ quality and enhancing compression/fit, we expect the data quality to improve. Training load and fatigue indicators provided important data, but these could be expanded by incorporating AI/machine learning to improve accuracy, personalizing data to the wearer, and extracting other valuable features (feature extraction). In addition, obtaining temporal data (data gathering over a period of time) was outside of the scope of this initial testing but will be performed in the future to study performance improvement and fatigue onset. Finally, this report describes our pilot results with Parkinson’s Disease patients, which showed promising results in monitoring initial time-to-onset commencement. In the future, we plan to conduct a clinical trial on a significantly larger scale (potentially, a multi-center trial), which will provide us with the ability to calculate the prediction performance with high reliability.

## 5. Conclusions

In conclusion, we designed smart textiles with embedded, integrated electrophysiologic, biochemical, and motion sensors that feed into enhanced, AI-enabled, medically oriented data analysis systems. We envision the design of a wearable garment that integrates IMUs for detecting velocity, acceleration, and motion of gait and surface sEMG sensors to measure muscular activity. Detectable changes in activity coupled with measurements of time-to-onset could reveal a previously unexplored opportunity to design haptic interventions for FOG prediction and fall alerts.

## Data Availability

The data supporting this article have been included in the manuscript text. Data collected from human participants, described in Figure 2, Figure 3 and Figure 4, are not available for confidentiality reasons.
